# Relationship between financial inclusion and carbon emissions: International evidence

**DOI:** 10.1016/j.heliyon.2023.e16472

**Published:** 2023-05-23

**Authors:** Shahzad Hussain, Muhammad Akbar, Raazia Gul, Syed Jawad Hussain Shahzad, Nader Naifar

**Affiliations:** a Department of Business Administration, Rawalpindi Women University, Pakistan; b Economics, Finance and Entrepreneurship Department, Aston Business School, Aston University, UK; c Faculty of Management Sciences, Shaheed Zulfikar Ali Bhutto Institute of Science & Technology, Karachi, Pakistan; d Montpellier Business School, France; e Imam Mohammad Ibn Saud Islamic University (IMSIU), Riyadh, Saudi Arabia

**Keywords:** GHG emissions, Inclusive financial system, EKC Hypothesis, Panel data

## Abstract

The nexus between financial inclusion and carbon emissions is becoming an increasingly important topic, given the augmented awareness of the negative impacts of climate change and carbon emissions on the environment and human health. In this study, we examine the impact of financial inclusion on carbon emissions using the STIRPAT framework for 102 countries from 2004 to 2020. We measure financial inclusion as a composite index, using principal component analysis (PCA) from five financial inclusion proxies. Our robust panel regression estimations suggest an N-Shaped relationship between financial inclusion and carbon emissions. The N-shaped Environmental Kuznets Curve (EKC) implies that the impact of financial inclusion on carbon emission is nonlinear and changes from an inverted U-shaped to a U-shaped. This finding is strong in developing countries and weak in advanced countries. It is also robust across our two normalized measures of financial inclusion as well as across different estimation techniques. These findings suggest adapting a universal environmental strategy that enhances financial inclusion through strong and accessible financial systems, particularly for low-income countries. Our results further suggest that government authorities and policymakers need to develop well-directed and inclusive financial policies that consider the varying levels of governance, regulations, and income across countries.

## 1 Introduction

Global warming and climate change have been the leading topics of worldwide discourse, as evidenced by the exponential growth in academic literature, media coverage, political dialogues, and legal and regulatory responses [1]. However, the continued rise in carbon dioxide (CO_2_) emissions, the cause of environmental degradation, jeopardizes the entire global climate system [2]. For example, the continued growth in carbon emissions may lead to a rise of 4 °C in average global temperatures that would have significant negative implications for economic, social, and environmental sustainability.^1^ Therefore, despite not being considered an air pollutant, CO_2_ is a major environmental threat due to its contribution to global warming [3]. Consequently, global efforts have been in place to improve environmental quality through carbon emissions addressing climate change challenges for sustainable development [4]. The Paris Agreement, which has 193 signatory parties, sets the goal of not letting the mean global temperatures increase by 1.5–2 °C. Achieving this goal requires significantly reducing environmental degradation by reducing carbon emissions [5].

Carbon dioxide emissions significantly contribute to climate change and have increased globally over the past few decades. Emissions trends vary between developed and developing countries, however. In recent years, emissions have started declining in developed countries due to the shift towards renewable energy sources, energy efficiency measures, and declining industrial output. For example, in the European Union, CO2 emissions have fallen by nearly 20% since1990,^2^ while in the United States, emissions have fallen by approximately 10% over the same period.^3^ On the other hand, emissions in developing countries have been increasing rapidly, driven by growing populations, urbanization, and rapid industrialization. For example, China has become the largest emitter of CO2 globally, with significantly growing emissions.^4^ India, Indonesia, and other countries in Southeast Asia have also seen a significant increase in emissions over the past few decades.^5^ However, there is a growing recognition of the need to reduce emissions, and many countries are starting to take action. For example, China, India, and other countries have set targets to increase the use of renewable energy and reduce their emissions.

CO_2_ emissions are deemed the biggest threat to achieving sustainable development, driving researchers' interest in finding the determinants of environmental quality [6]. Whether continuous economic development sufficiently covers environmental costs or degrades the environment is of paramount importance, which the extant literature has addressed extensively [2,7]. The relationship between trade openness [8], industrialization [5], foreign direct investment [9], and environmental taxes [10] in reducing environmental degradation has also been empirically assessed. Financial growth fosters the financial sector's progression [[11], [12], [13]]. Many researchers have empirically investigated its relationship with carbon emissions [14,15], and the relevant empirical literature on this nexus is still evolving. The lack of relevant studies is mainly due to the dearth of data on the financial inclusion index [16,17].

Financial inclusion refers to accessing an inclusive spectrum of financial instruments and services by all individuals and businesses (World Bank, 2022).^6^ Financial inclusion plays an influential role in achieving economic growth as it ensures capital creation, enhanced understanding of investment activities, and efficient allocation of resources [18]. Furthermore, as an accurate measure of financial assets, financial inclusion is argued to help reduce ecological damage and hence reduce the rate of environmental degradation [19], and improve environmental quality through R&D initiatives, expansions, and foreign direct investments [20]. In addition, financial development allows firms and governments to reduce carbon emissions and enhance environmental quality by increasing affordability to adopt environmentally efficient technologies [16].

Though empirical literature and theoretical underpinning suggest a positive contribution of financial inclusion in building environmental sustainability, the extant literature also provides contradictory opinions and empirical evidence. Financial inclusion may increase environmental degradation by fostering industrial activities through cheaper financing resulting in higher carbon emissions [21]. [22] argued that countries at different economic and financial development phases might experience different levels of carbon emissions, suggesting a nonlinear relationship. Since [23] developed EKC, the scant literature attempts to investigate the nonlinear nexus of financial development and climate change [19,24,25]. In this line of argument, financial inclusion damages the environment during the first phase of the financial development that reverses in the later stage as economic growth benefits environmental preservation. In other words, an inverted U-shaped nexus was observed between greenhouse gas (hereafter GHG) emissions and financial inclusion [10,19].

The objectives of our study are as follows:

i) To examine the financial inclusion-carbon emissions nexus using the STIRPAT framework while considering the stage of financial development.
ii) To suggest a universal environmental strategy to enhance financial inclusion in low-income countries by improving their financial systems.
iii) To provide insights into the potential for financial inclusion to achieve environmental sustainability and reduce environmental degradation.

The motivation for this study stems from the global concern over environmental degradation and the threat posed by increasing CO2 emissions to the entire global climate system. The Paris Agreement, signed by 193 countries, sets a goal of not letting the global temperature rise by more than 2 °C and striving to reduce environmental degradation and carbon emissions for sustainable development. Although the literature on the relationship between financial inclusion and CO2 emissions has been the subject of empirical investigation for quite some time, there is a lack of data on the financial inclusion index. Despite the theoretical and empirical evidence suggesting a positive contribution of financial inclusion to environmental sustainability, the existing literature still provides conflicting opinions and empirical evidence. The present study aims to address the mixed findings on this association, filling the gap in the literature and making various practical implications for policymakers and regulators.

Given the mixed empirical findings on the association between financial inclusion and GHG emissions, the present study has four unique contributions. *First*, we extend the EKC hypothesis proposing an N-shaped relationship between financial inclusion and GHG emissions in line with the earlier work of [26]. It is justified since the EKC hypothesis fails to hold globally as increased income may positively correlate with carbon emissions after a certain income level [27]. Ref. [26] explained that it could not be implied that a high-income country would have lower GHG emissions as the scale effect may exceed the technical effect resulting in technical obsolescence. Ref. [26] further argued that technological advances might not solve every environmental problem. With scarce resources, the environment may not sustain high-income societies after a certain point. For empirical analysis, we extend this argument and posit the same relationship between financial inclusion and GHG emissions. Hence, consistent with [26], we postulate the nexus between the inclusive financial system and GHG emissions to exhibit an inverted U-shaped EKC pattern in the short to medium term and an N-shaped pattern in the long term. Therefore, the present study is unique as it tests the N-shaped nexus between GHG emissions and financial inclusion.

*Second*, the financial sector’s development has varying effects on carbon emissions depending on the level of economic and technological development [28]. Developed countries have solid environmental protection mechanisms; hence, businesses in such countries are inclined to invest in technological innovation rather than scale expansion [14,29]. Existing literature indicates a nonlinear correlation between financial advancement and greenhouse gas emissions, which can be explained by robust governance systems and stringent environmental policies focused on sustainable growth [1,19]. On the contrary, developing countries have weak environmental protection regulations that incentivize high growth and economic development, expanding production capacity through affordable credit facilities [30,31,32]. We investigate the nexus between financial inclusion and GHG emissions, postulating an inverted U-shaped EKC pattern in the short to medium term and an N-shaped nexus in the long term that may vary between developed and developing countries.

*Third*, we use a large sample of 102 countries over an extended period covering 2004 to 2020, an improvement over [19]. Finally, the present study employs Driscoll–Kraay standard errors (D-K) for estimation and alternate advanced statistical techniques, including Feasible generalized least squares (FGLS) and Difference GMM validating the robustness of the results. Our panel data regression results show that the relationship is nonlinear and N-Shaped, implying that the impact of financial inclusion on carbon emission changes from an inverted U-shape to a U-shaped one. This N-shaped relationship is statistically strong in developing countries compared to advanced countries and is robust across different measures of financial inclusion and estimation techniques.

The above discussion stresses the need to study the financial inclusion-carbon emission nexus because it sheds light on how financial systems can play a role in mitigating climate change. Examining this relationship can help identify ways to encourage investment in low-carbon technologies and infrastructure. This can further lead to the development of innovative financial products and services that can mobilize investment and support the transition to a low-carbon economy. Moreover, climate change poses a risk of stranded assets, investments that become valueless due to environmental or regulatory changes. Our study can help identify ways to close the financing gap for low-carbon projects and support their development and scaling.

The remainder of the paper is organized as follows. Section 2 presents the literature review, while Section 3 describes the research methodology. Section 4 describes the data, discusses the main findings, and illustrates the robustness check. Finally, section 5 concludes the study and offers some implications.

## 2 Literature review

### 2.1 Positive association between financial inclusion and environmental quality

The extant literature acknowledges the positive role of the financial sector in combating climate change to achieve sustainable and inclusive economic development [33]. The encouraging contribution of financial sector growth in reducing carbon emissions has been documented by earlier studies, such as [[34], [35], [36]], suggesting that financial development-induced R&D may reduce environmental damage enabling the adoption of modern, efficient, clean and green technologies. Consistent with the significant role of economic development in eradicating carbon emissions, empirical research on the linear relationship between GHG emissions and inclusive financial growth suggests that increased financial inclusion reduces environmental degradation [12]. Analysing a sample of 119 countries from 1980 to 2006, Ref. [37] reported that financial intermediation, i.e., commercial finance, positively affected the amount of renewable energy produced. Ref. [38] reported similar findings for their sample of 33 OECD countries grouped into lower and higher globalized economies. Financial inclusion reduces CO2 emissions in China through two channels; increasing vegetation coverage that raises carbon sequestration capacity and enhancing industrial structure because of financial inclusion [3]. Likewise [12], found that financial inclusion reduced CO2 emissions in China in the short and long run from 1995 to 2019 [39]. also supported the notion that carbon emissions were reduced with increased financial inclusion in Ghana.

### 2.2 Negative association between financial inclusion and environmental quality

A plethora of studies, such as [32,40], and [41], empirically supported the notion that financial development degrades environmental sustainability. Ref. [16] investigated the causal impact of an inclusive financial system on CO2 emissions of 31 Asian countries from 2004 to 2014 using Driscoll-Kraay standard error models. Their empirical findings support the proposition that an inclusive financial system threatens environmental sustainability due to increased carbon emissions [9,10,18,42]. reveal the same negative association of financial inclusion with environmental quality for their samples of OECD countries, Eurozone, China, and Next Eleven countries respectively. Ref. [43] observed the same relationship for their sample of South Asian countries and attributed this to a lack of regional policy coherence and coordination. However, it is argued that the said negative association does not imply reducing financial inclusion but rather aligning its initiatives with a country's environmental policy. Ref. [44] investigated the short- and long-term association between financial development and carbon emissions in China from 1980 to 2014. They suggested that financial development significantly impacts CO2 emissions in China in both the short- and long run. Ref. [45] employed the ARDL approach and found similar positive results in Nigeria for their sample period of 1971–2010. Ref. [46] applied the CS-ARDL model and found similar findings about the BRICS economies over the sample period from 1990 to 2020. Ref. [47] recently employed the PGM-ARDL model and reported a positive association between environmental degradation and financial inclusion in the ASEAN region from 2000 to 2019.

The extant literature also provides empirical evidence in support of the nonlinear financial growth and environmental deprivation relationship in line with the EKC hypothesis that supports a U-shaped relationship [29,30,41,48,49]. For instance, Ref. [50] reported a nonlinear association of development related to the financial system and GHG emissions for 25 OECD countries from 1971 to 2007. Ref. [51] supported the EKC hypothesis using advanced statistical methods, including CUP-BC and CUP-FM, for their sample of 17 APEC countries from 1990 to 2016. Using data from 102 countries over a sample period from 2004 to 2014, Ref. [19] examined the connection of carbon emissions to development related to financial inclusion within the EKC framework and reported the inverted U-shaped relationship. Using 74 heterogeneous financial economies and region over the period of 2004–2020, Ref. [1] supported the EKC based inverted U-shaped impact of inclusive financial system on CO2 emissions. However, this non-linear nexus of inclusive financial system with CO emission varies across level of economic development and regions.

Since the seminal work by Ref. [23] on EKC, the N-shaped nexus has been empirically investigated and documented for economic development and environmental degradation [[52], [53], [54], [55], [56]]. However, the N-shaped nexus between financial inclusion and GHG emissions is unexplored. Therefore, our study aims to contribute to the literature on the N-shaped relationship between GHG emissions and inclusive financial development for developed and developing countries. We posit that this nexus may follow an inverted U-shaped EKC pattern in the short to medium term and an N-shaped nexus in the long term, which may further vary for developed and developing countries. Based on the literature review, we formulate the following hypothesis:

H1 There exists an N-shaped relationship between financial inclusion and carbon emissions

### 2.3 Literature gap

The above discussion shows that the empirical findings on the linear and nonlinear relationship between financial inclusion and environmental sustainability are mixed and inconclusive. Primarily, we identify four gaps in the extant literature the present study addresses.

*First*, the existing literature primarily focuses on the linear and U-shaped relationships between financial inclusion and environmental degradation. In contrast, our study aims to extend the Environmental Kuznets Curve hypothesis by exploring the N-shaped relationship. This means that the study seeks to examine if there is a threshold level of financial inclusion beyond which an increase in financial inclusion results in an increase in GHG emissions.

*Second*, as empirical evidence suggests, financial inclusion's impact on environmental degradation may vary per the country's economic development and quality of governance. Our study seeks to examine this relationship by dividing the sample into two subsamples - developed and developing countries - and by controlling for the quality of management.

*Third*, our study seeks to improve the existing literature by using a more extensive sample period (2004–2020) and a larger sample size (102 countries). The study also addresses the limitations of [19], who used a smaller sample period for 103 countries.

Fourth, our work relates to a few studies investigating the nexus of CO2 emissions and inclusive financial systems using advanced statistical techniques, including Driscoll–Kraay standard errors (D-K), Feasible generalized least squares (FGLS), and Difference GMM. This allows for a more robust examination of the relationship and helps address the limitations of previous studies that may have used simpler statistical models. We aim to contribute to the literature by addressing these literature gaps and better understanding this nexus.

## 3 Data and methodology

### 3.1 Sample details

We utilized a panel dataset from the World Development Indicators (WDI) covering 102 countries from 2004 to 2020 for our empirical analysis. However, the data period was restricted due to inadequate availability, particularly for the sub-components creating the financial inclusion index. The sample countries are mentioned in Table A1 in the appendix. We are following the line of reasoning of [19] that non-linearity between financial sector development and carbon emissions exist due to solid governance mechanism and strict environmental regulations to ensure sustainable development. In addition, extant literature supports the notion that developed countries have solid environmental protection mechanisms; hence, businesses in such countries are inclined to invest in technological innovation rather than scale expansion [1,14,29]. On the contrary, developing countries have weak environmental protection regulations that incentivize high growth and economic development, expanding production capacity through affordable credit facilities (Jiang & Ma, 2019). Hence, we have divided the selected 102 countries into two subsamples based International Monetary Fund’s classification of income groups by following the approach of [57,58]. Specifically, we categorized 65 countries as developed nations, comprising high-income and upper-middle-income countries. The remaining 37 countries were classified as developing countries, including lower- and lower-middle-income ones.

### 3.2 Operationalization of variables

To conduct empirical analysis, we categorized our study variables into three sets: independent, dependent, and control. Our independent variable is the financial inclusion index (FI), while the dependent variable is carbon emission (CO2). Financial inclusion is typically measured by five dimensions: availability, affordability, quality, usage, and penetration of financial services (Sarma, 2015). However, recent research by Refs. [16,19] suggests that the availability and usage of formal financial services are critical components of an inclusive financial system. Accordingly, we adopted five proxies to construct a composite measure of financial inclusion, namely the number of commercial bank branches per 100,000 adults, the number of ATMs per 100,000 adults, institutions of commercial banks, institutions of commercial banks, outstanding bank loans, and bank deposit amount, consistent with [16,59]. The chosen proxies cover two key aspects, where the first three relate to the banking sector's availability, and the last two pertain to the banking system's usage. Before conducting principal component analysis (PCA), we normalize the proxies using two techniques, namely Z-score and Min-Max, as recommended by Ref. [60].

Principal Component Analysis (PCA) is a standard method to create indices, considered effective for extracting hidden features and relationships. It removes excess information reducing data dimensionality in developing composite indicators [61]. Before applying PCA, we determine the chosen proxies to be valid for constructing the financial inclusion index. For this purpose, we conduct Kaiser-Meyer-Olkin (KMO) and Bartlett’s test for Sphericity. If Bartlett’s test is statistically significant, then factor analysis is better. Similarly, factor analysis is considered appropriate if the KMO test > 0.5, as it ranges between 0 and 1. Both of these tests support the use of PCA, and these results are reported in Table 1.

**Table 1:** Results of Bartlett and KMO tests for sphericity & sampling adequacy – Financial inclusion index.

	Bartlett Results		KMO Results
	Chi-Square	Df	
Z-Score	1791.318***	10	0.7110
Min-Max Score	1778.865***	10	0.7060

*Notes*. Statistical significance is denoted by ***, **, and * at 1, 5, and 10%.

PCA analysis is performed in two steps. Firstly, the components are investigated to identify the ones that explain the variations in the variable of interest with the lowest pairwise correlations. Secondly, the FI index is constructed based on the components identified in step one (eigenvalues greater than 1). Table 2 provides the cumulative variations of each element from our PCA analysis.

**Table 2:** PCA analysis of financial inclusion variables.

Variable	PC1	PC2	PC3	PC4	PC5
Financial Inclusion Indicators based on Z-Score					
Commercial banks' branches per 100,000 adults	0.4919	0.1516	−0.0007	−0.8172	−0.2592
ATMs per 100,000 adults	0.4943	0.1345	−0.6655	0.1584	0.5192
Outstanding loans from commercial banks	0.5352	−0.0813	−0.0129	0.5173	−0.6627
Outstanding deposits with commercial banks (% of GDP)	0.4764	−0.2391	0.6959	0.092	0.4723
Institutions of commercial banks	0.0173	0.9461	0.2695	0.1761	0.0302
Financial Inclusion Indicators based on Min Max-Score					
Commercial banks' branches per 100,000 adults	0.4914	0.1799	−0.0184	−0.8026	−0.2859
ATMs per 100,000 adults	0.4968	0.1142	−0.6687	0.161	0.5167
Outstanding loans from commercial banks	0.5326	−0.1073	0.0045	0.533	−0.6486
Outstanding deposits with commercial banks (% of GDP)	0.476	−0.2597	0.6897	0.0473	0.4776
Institutions of commercial banks	0.0381	0.9358	0.2771	0.2088	0.05

Concerning the control variables, existing literature suggests that economic development has the potential to provide individuals and businesses with easier access to financial services, which can motivate firms to expand production and enable consumers to purchase energy-intensive electric appliances, resulting in increased use of fossil fuels and higher CO2 emissions [62]. Additionally, empirical studies have established that population growth can worsen the environmental quality and lead to higher per capita CO2 emissions [63]. Furthermore, industrialization is a significant contributor to CO2 emissions in the region, consistent with previous research on the negative impact of industrialization on environmental quality [26]. Similarly, the pollution havens hypothesis supports that trade openness exacerbates environmental degradation [64]. Considering the above discussion, we consider GDP per capita, population growth, industrialization, and trade openness as control variables for data analysis.

### 3.3 Statistical techniques for data analysis

This study utilizes a static and dynamic panel estimation method to explore the relationship between inclusive financial sector advancement and CO2 emissions, building on the benefits [1] recommended in a panel setting. This method presents various advantages. Firstly, it leverages the amalgamation of data across time and sections, allowing for more comprehensive insights. Secondly, it significantly diminishes the issue of individual heterogeneity that may arise in other forms of estimation. Lastly, it facilitates the detection of unobserved heterogeneity, which is unobservable otherwise.

We performed the data analysis in five steps. First, we estimate the descriptive statistics to identify the issue of outliers. In the second step, following [16], we perform the [65] to check the cross-sectional dependence and Wooldridge Test and Modified Wald tests for serial correlation within panels and group-wise heteroscedasticity, respectively. In 3rd step, we empirically tested the impact of financial inclusion, based on Z-score and mini-max, on CO2 emissions both in subsamples and the whole sample through [66] advance panel estimator and with robust standard error through the command of [67] to obtain compressive, complete and consistent regression coefficients even in the presence of heteroscedasticity, temporal and cross-sectional dependence [1]. In the 4th step, we test the causal impact of an inclusive financial system on carbon emissions through the FGLS model as a robustness check. FGLS is considered the most suitable to produce consistent and unbiased coefficients even in autocorrelation within-group and panel-wise heteroscedasticity to verify and validate the empirical findings. Finally, we test financial inclusion and carbon emission nexus in a dynamic panel framework through different GMM as robustness checks to validate the main findings.

### 3.4 Econometric models

We utilize stochastic impacts by regression on population, affluence, and technology (STIRPAT) as a theoretical base to examine the financial inclusion-CO_2_ emissions nexus [26]. Mathematically the STIRPAT model is given as follows:

(1) $$I_{it}=\delta_{0}+\delta_{1}P_{i,t}+\delta_{2}A_{i,t}+\delta_{3}T_{i,t}+\varepsilon_{i,t}$$

where $I_{it}$ represents environmental effects, *P*_*it*_, *A*_*it,*_ and *T*_*it*_ represent the population, affluence, and technology factors. The subscript *i* denotes a country, and *t* is time, i.e., year. We add financial inclusion (FI) to the STIRPAT model in equation (1). Consistent with extant literature, we include four control variables, including the log of GDP per capita, population growth (POPG), industrialization (IND), and trade openness (TO) to the STIRPAT model [4,16,68]. The baseline model is:

(2) $${CO2}_{i,t}=\alpha_{0}+\alpha_{1,i,t}{FI}_{i,t}+\alpha_{2,i,t}{FI}_{i,t}^{2}+\alpha_{3,i,t}{FI}_{i,t}^{3}+\sum_{t=1}^{t=n}\alpha_{i,t}{Controls}_{i,t}+\varepsilon_{i,t}$$

In Eq. (2), ${CO2}_{i,t}$ represents the log of CO2 emissions (metric tons per capita), ${FI}_{i,t}$ denotes the index for financial inclusion, whereas ${FI}^{2}$ and ${FI}^{3}$ denote the squared and cubic term of financial inclusion. The control variables are the log of GDP per capita, population growth, industrialization, and trade openness. EKC follows a different shape depending on the sign of the coefficients of our variables [26] that are outlined as follows:

a). An N-shaped environmental Kuznets curve (EKC) would result if α1 > 0, α2 < 0, and α3 > 0. b). An inverted N-shaped EKC would occur if α1 < 0, α2 > 0, and α3 < 0.
c). A U-shaped EKC would emerge if α1 < 0, α2 > 0, and α3 = 0.
d). An inverted U-shaped EKC would result if α1 > 0, α2 < 0, and α3 = 0.
e). A monotonically increasing linear nexus between financial inclusion and carbon emissions would arise if α1 > 0 and α2 = α3 = 0.
f). A monotonically decreasing linear association between financial inclusion and carbon emissions would occur if α1 < 0 and α2 = α3 = 0.
g) No significant nexus between financial inclusion and carbon emissions would be evident if α1 = α2 = α3 = 0.

## 4 Findings

### 4.1 Descriptive statistics

The summary statistics of all variables are reported in Table 3. The average and volatility of carbon emission (M = 4.148, SD = 4.550) are relatively lower compared to Ref. [19], implying a reduction in average carbon emission levels across the sample countries over the recent years' data included. The summary statistics of five proxies of financial inclusion (FI) reveal that mean values of commercial bank branches per 100,000 users, ATMs per 100,000 users, and Institutions of commercial banks are greater than the average values of these proxies reported in Ref. [16] for Asia region. Moreover, the outstanding deposits and loans as a percent of GDP are greater than those reported by Ref. [19], suggesting that the selective sample countries have better financial inclusiveness.

**Table 3:** Descriptive statistics of the selected variables for the entire sample.

Variables	Obs.	Mean	Std. Dev.	Min	Max
Carbon Emission (CO_2_)	1734	4.148	4.550	0.0047	29.623
CBB	1701	18.215	16.423	0.300	110.94
ATM	1653	45.453	47.651	0.000	324.172
OLC	1706	50.764	42.106	1.266	304.575
ODC	1677	61.394	64.037	2.663	770.258
ICB	1757	45.692	102.221	1.000	1249.000
Economic Growth (GDP)	1734	24.066	2.332	17.678	30.32
Population Growth (POP)	1734	1.314	1.247	−2.258	6.559
Industrialization (IND)	1734	26.394	13.643	3.15	87.797
Trade openness (TO)	1734	98.165	59.692	0.167	442.62

*Notes*. This table reports the descriptive statistics of the variables. Std. Dev. Min and Max stand for standard deviations, minimum and maximum, respectively. Carbon Emission (CO2) is CO2 in MC per Capita; CBB is commercial banks' branches per 100,000 adults; ATM stands for ATMs per 100,000 adults; OLC is outstanding loans from commercial banks (% of GDBP); ODC is outstanding deposits with commercial banks (% of GDP); ICB is institutions of commercial banks; Economic Growth (GDP)is the log of GDP per capita; Population Growth (POP) is the percentage change in Population; Industrialization (IND) is the value added as a percentage of GDP by industry; Trade openness (TO) is measures as export plus imports divided by GDP.

**Fig. 1:**
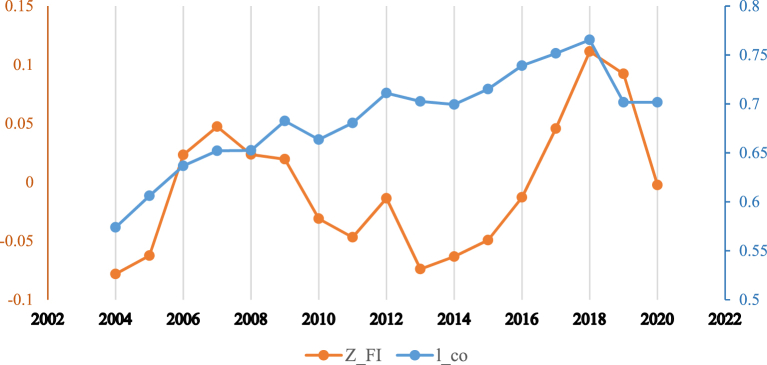
The annual cross-sectional averages of financial inclusion (FI) and log of carbon emission (co) for the full sample. Source: Author’s calculations based on sample data used for this study.

**Fig. 2:**
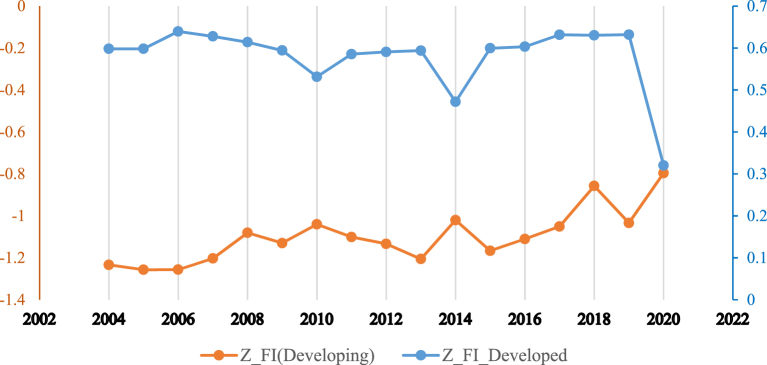
The annual cross-sectional averages of financial inclusion for developed and developing countries samples. Source: Author’s calculations based on sample data used for this study.

**Fig. 3:**
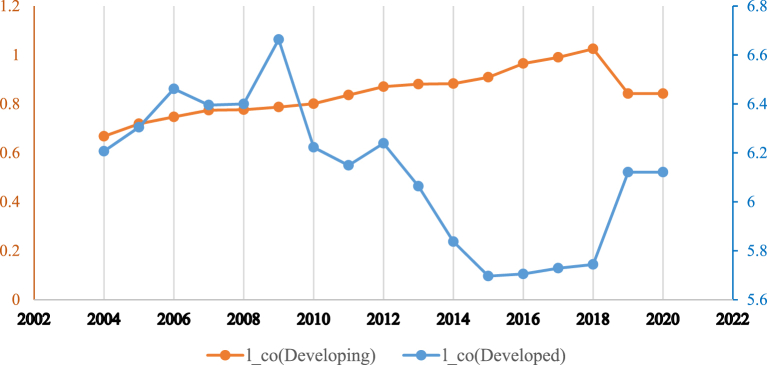
The annual cross-sectional averages of log of carbon emissions for developed and developing countries samples. Source: Author’s calculations based on sample data used for this study.

**Fig. 4:**
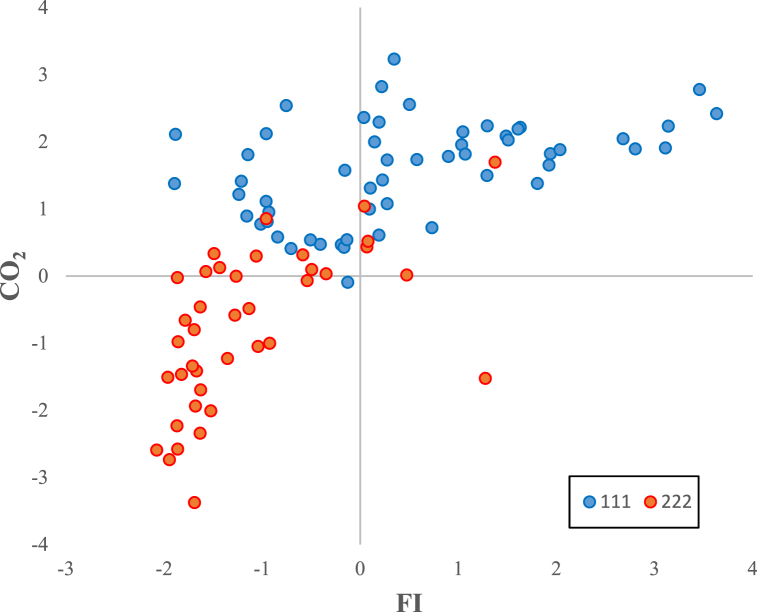
Scatter diagram of annual cross-sectional averages of financial inclusion and carbon emissions. Note. This figure shows the scatter plot of annual cross-sectional averages of financial inclusion scores and log of carbon emissions. The blue (orange) dots represent the cross-sectional averages of developed (developing) countries. Source: Author’s calculations based on sample data used for this study. (For interpretation of the references to colour in this figure legend, the reader is referred to the Web version of this article.)

Fig. 1 shows the relationship between the inclusive financial system and carbon emissions in the whole sample. It can be observed that the relationship has a non-linear trend. Fig. 2 shows that developed countries enjoy greater financial inclusiveness vis-à-vis developing countries. However, Fig. 3 reveals that greenhouse gas emissions are higher in developing economies, which could be due weak governance structure of low-income countries. Moreover, it can be observed from Fig. 4 reveals that there exists a non-linear N-shaped financial inclusion and CO2 emission nexus.

The results of cross-sectional dependence (CD) tests based on [65] are reported in Table 4. The CD test is appropriate when cross-sections are larger than the length of the time series, such as the present where N = 102 > T = 17. Table 4 reveals statistically significant at one percent, hence rejecting the null hypothesis of cross-sectional independence. Therefore, the test results suggest the presence of cross-sectional dependency amongst the variables across all the countries. Furthermore, the results of the [69] test and the Modified Wald test by Ref. [70] testing serial correlation and heteroscedasticity assumptions of regression are reported in Table 5. The test results suggest the existence of serial correlation, while the presence of heteroscedasticity is confirmed through the Modified Wald test.

**Table 4:** Results of Pesaran (2004) cross-section independence tests.

Variables	CD-test statistic
CO2	13.69 ***
FI (Z-Score)	22.60***
FI (Min-Max Score)	21.507***
GDP	236.88 ***
POPG	20.67 ***
IND	35.68***
TO	26.42***

*Note*. *** indicates rejection of the null hypothesis of cross-sectional independence at a 1% significance level.

**Table 5:** Diagnostic test results.

Test	Error Process	Test statistic
Financial Inclusion Indicators based on Z-Score		
Modified Wald (χ 2) H	Heteroscedasticity	201,000***
Wooldridge Test (F-test)	Serial Correlation	14.61***
Financial Inclusion Indicators based on Min Max-Score		
Modified Wald (χ 2) H	Heteroscedasticity	270,000***
Wooldridge Test (F-test)	Serial Correlation	5.562**

*Note*. *** and ** indicate rejection of the null hypothesis at 1% and 5% significance levels, respectively.

### 4.2 Empirical analysis

We investigate the relationship between financial inclusion and carbon emissions through advanced statistical methods utilizing subsamples and the full sample of developed and developing countries to produce efficient and consistent estimates accounting for cross-sectional dependency, serial correlation within panels, and group-wise heteroskedasticity. We normalize the financial inclusion proxies using Z-score and Min-Max techniques consistent with [16] and then used the normalized values of proxies in PCA analysis to construct separate financial inclusion indices – one with Z-score normalization and one with Min-Max normalization. The regression results of the Z-score normalized PCA financial inclusion index are reported in Table 6. The results in Table 6 show that the D-K regression coefficients of the linear and cubic terms of financial inclusion are positive. In contrast, the quadratic form of financial inclusion is negative (*α*_1_ > 0, *α*_2_ < 0, and *α*_3_ > 0), which suggests its N-shaped association with carbon emissions. The findings support the notion that N-shaped EKC holds in the long run.

**Table 6:** The estimation results of the N-shaped relationship between Financial Inclusion (Z-Score) and Carbon Emission.

Variables	Full Sample			Developed Countries			Developing Countries		
	D-K	FGLS	GMM	D-K	FGLS	GMM	D-K	FGLS	GMM
L.CO_2_			0.9550***			0.7680***			0.8810***
			(0.0078)			(0.0057)			(0.0122)
FI	0.6020***	0.6021***	0.0986***	0.3440***	0.3440***	0.0691***	0.3700***	0.3700***	0.0759**
	(0.0232)	(0.0211)	(0.0105)	(0.0268)	(0.0208)	(0.0087)	(0.0606)	(0.0781)	(0.0324)
FI^2^	−0.1790***	−0.179***	−0.0287***	−0.0175	−0.0175	−0.0203***	−0.0604*	−0.0604*	−0.0252***
	(0.0064)	(0.0123)	(0.0030)	(0.0140)	(0.0139)	(0.0028)	(0.0298)	(0.0361)	(0.0082)
FI^3^	0.0191***	0.0191***	0.0026***	−0.0022	−0.0022	0.0027***	0.0536*	0.0536**	0.0087*
	(0.0007)	(0.0025)	(0.0003)	(0.0019)	(0.0024)	(0.0003)	(0.0265)	(0.0239)	(0.0052)
GDP	0.1060***	0.1060***	0.0175***	0.0611***	0.0611***	0.0218***	0.0960***	0.0960***	−0.0011
	(0.0081)	(0.0113)	(0.00244)	(0.0086)	(0.0101)	(0.0028)	(0.0285)	(0.0205)	(0.0031)
POP	−0.1270***	−0.1270***	0.0178***	−0.0002	−0.0002	−0.0098***	−0.2990***	−0.2990***	−0.0137**
	(0.0244)	(0.0173)	(0.0026)	(0.0128)	(0.0146)	(0.0015)	(0.0756)	(0.0346)	(0.0063)
IND	0.0386***	0.0386***	0.0039***	0.0259***	0.0259***	0.0068***	0.0333***	0.0333***	0.0044***
	(0.0008)	(0.0016)	(0.0006)	(0.0007)	(0.0015)	(0.0006)	(0.0016)	(0.0028)	(0.0005)
TO	0.0051***	0.0051***	−0.0004***	0.0038***	0.0038***	0.0010***	0.0043***	0.00433***	0.0002
	(0.0004)	(0.0004)	(0.0004)	(0.0003)	(0.0004)	(0.0000)	(0.0009)	(0.0007)	(0.0001)
Constant	−2.7760***	−2.7760***	0.4470***	−1.1990***	−1.1990***	−0.4760***	−2.8110***	−2.8110***	0.0300
	(0.2250)	(0.2860)	(0.0598)	(0.2310)	(0.2570)	(0.0657)	(0.6500)	(0.4920)	(0.0859)
R-squared	0.729			0.532			0.676		
Wald (Prob > chi2)		0.000	0.000		0.000	0.000		0.000	0.000
F-Stats(P Value)	0.000			0.000			0.000		
AR(1)			0.0002			0.005			0.006
AR(2)			0.553			0.537			0.356
Hansen test (p value)			0.293			0.434			0.830
No of Countries	102	102	102	65	65	65	37	37	37

*Note:* The standard errors are shown in brackets. ***, **, and * indicate significance at 1%, 5% and 10% level, respectively. D-K: Driscoll and Kraay (1998) standard errors for coefficients were estimated by pooled Ordinary Least Squares/Weighted Least Squares regressions. FI, FI^2^ and FI^3^ denote composite financial inclusion index constructed by performing PCA on financial indicators normalized by z-score, the squared FI and cubic FI, respectively.

The existence of N-shaped-based inclusive financial inclusion and carbon emissions in the whole sample support the notion that accessible financial resources with the lower cost of borrowing for individuals and businesses are more favorable in the short run and would positively contribute to environmental quality. However, beyond a threshold level, the environmental quality could not be sustainable due to the rapid expansion of production capacity at the corporate level and the upsurge in energy-intensive home appliances at individual levels as a result of accessible credit at a lower cost of capital. Consequently, leading to technical obsolescence, as the scale effect might surpass the technique effect. Hence, the results reveal that too much financial inclusiveness deteriorates the long-term environmental quality. The results contradict [19], as this study established a non-linear EKC- based financial inclusion and carbon emissions in 103 countries.

The analysis of subsamples also established a statistically significant and robust N-shaped nexus between inclusive finance and CO2 emissions in low-income markets. These findings confirm that developing economies are under extreme pressure to produce more output via capacity expansion of production plants to achieve higher economic growth, further exacerbating environmental deterioration. Secondly, developing countries have limited financial capital due to lower saving rates and hence more focused on industrial development and expansion to achieve rapid economic growth. Thirdly, a weak governance structure hinders the execution of the green project for sustainable development in developing countries. However, the results reveal weak N-shaped EKC-based financial inclusion and carbon emission relationship in developed markets. The results are consistent with the notion that adequate credit facilities at a lower cost of borrowing and a strong and transparent governance structure encourage individuals and businesses to invest in green and technologically intensive projects to increase overall output and contribute to sustainable development. Likewise, Fig. 4 presents the graphic representation of financial inclusion and carbon emission in subsamples. The results depict the noticeable difference in the N-shaped relationship in developed vis-à-vis developing countries. The results contradict the findings of [1]. This study concluded the inverted U-shaped EKC-based inclusive financial system relationship with CO2 emissions. Our results are robust across the financial inclusion index constructed using PCA with Min-Max normalized proxies (Table 7).

**Table 7:** The estimation results of the N-shaped relationship between Financial Inclusion (Mini-Max) and Carbon Emission.

Variables	Full Sample			Developed Countries			Developing Countries		
	D-K	FGLS	GMM	D-K	FGLS	GMM	D-K	FGLS	GMM
L.CO_2_			1.0170***			0.7980***			0.8290***
			(0.0060)			(0.0027)			(0.0144)
FI	0.6080***	0.6080***	0.0591***	0.3480***	0.3480***	0.0431***	0.2670***	0.2670***	0.1210***
	(0.0170)	(0.0212)	(0.0080)	(0.0251)	(0.0210)	(0.0042)	(0.0489)	(0.0752)	(0.0277)
FI^2^	−0.1850***	−0.1850***	−0.0355***	−0.0229	−0.0229	0.0120***	−0.0681**	−0.0681**	−0.0085**
	(0.0081)	(0.0123)	(0.0028)	(0.0146)	(0.0139)	(0.0013)	(0.0320)	(0.0322)	(0.0042)
FI^3^	0.0199***	0.0199***	0.0037***	−0.0016	−0.0016	0.0017***	0.0889***	0.0889***	0.0115**
	(0.0010)	(0.0025)	(0.0003)	(0.0019)	(0.0024)	(0.0001)	(0.0153)	(0.0217)	(0.0055)
GDP	0.0972***	0.0972***	0.0069***	0.0604***	0.0604***	0.0193***	0.0734***	0.0734***	0.0277***
	(0.0074)	(0.0114)	(0.0024)	(0.0079)	(0.0101)	(0.0016)	(0.0188)	(0.0205)	(0.0044)
POP	−0.1290***	−0.1290***	−0.0215***	−0.0011	−0.0011	−0.0047***	−0.2810***	−0.2810***	−0.0728***
	(0.0254)	(0.0171)	(0.0032)	(0.0123)	(0.0146)	(0.0017)	(0.0486)	(0.0338)	(0.0091)
IND	0.0395***	0.0395***	−0.0014***	0.0263***	0.0263***	0.0048***	0.0338***	0.0338***	0.0059***
	(0.0006)	(0.0016)	(0.0004)	(0.0009)	(0.0015)	(0.0001)	(0.0013)	(0.0028)	(0.0005)
TO	0.0051***	0.0051***	0.0000	0.0039***	0.0039***	0.0008***	0.0040***	0.0040***	0.0009***
	(0.0003)	(0.0004)	(0.0000)	(0.0003)	(0.0004)	(0.0000)	(0.0006)	(0.0007)	(0.0001)
Constant	−2.5830***	−2.5830***	−0.1800***	−1.1970***	−1.1970***	−0.3840***	−2.3100***	−2.3100***	−0.7290***
	(0.1850)	(0.2870)	(0.0619)	(0.1950)	(0.2590)	(0.04150)	(0.4530)	(0.4930)	(0.1090)
R-squared	0.731			0.529			0.690		
Wald (Prob > chi2)		0.000	0.000		0.000	0.000		0.000	0.000
F-Stats(P Value)	0.000			0.000			0.000		
AR(1)			0.002	0.002		0.006			0.004
AR(2)			0.169	0.169		0.544			0.269
Hansen test (p value)			0.117	0.117		0.281			0.478
No of Countries	102	102	102	65	65	65	37	37	37

*Note:* The standard errors are shown in brackets. ***, **, and * indicate significance at 1%, 5% and 10% level, respectively. D-K: Driscoll and Kraay (1998) standard errors for coefficients were estimated by pooled Ordinary Least Squares/Weighted Least Squares regressions. FI, FI^2^ and FI^3^ denote composite financial inclusion index constructed by performing PCA on financial indicators normalized by the min-max technique, the squared FI and cubic FI respectively.

Concerning the control variables, economic growth is positively associated with CO2 emission, suggesting that rapid economic development further deteriorates environmental quality. The results are consistent with the notion that economic progress leads to an accessible financial system for individuals and businesses. Consequently, the available lower cost of credit upsurge the individual consumption pattern and engages the enterprises in production expansion, further damaging the environmental quality [62]. Similarly, massive industrialization has a statistically significant positive impact on CO2 emissions. This aligns with the notion that industrial development exacerbates environmental quality [26]. Likewise, the impact of positive and statistically significant findings is consistent with the pollution heaven hypothesis [64]. However, the effect of population growth is a statistically significant negative coefficient, suggesting that population growth does not always deteriorate environmental quality. The findings are consistent with [71].

Overall, our findings are robust with the theoretical notion that N-shaped EKC holds. An increase in financial inclusion after a certain threshold may result in a positive relationship between environmental degradation and financial inclusion [27]. Moreover, the weak N-shaped relationship may be attributed to the notion that due to solid governance mechanisms and strict environmental regulations underlying environment, social, and governance (ESG), developed countries ensure sustainable development (see, e.g. Ref. [19]). However, considering the weak governance structure of developing countries, this relationship is N-Shaped.

Further, the results support the previous findings of weak N-shaped nexus in the subsample of developed countries. Overall, in the long run, the findings negate the U-shaped EKC hypothesis consistent with [27] and support the notion of the N-shaped EKC. Ref. [56] explain that the N-shaped relationship exists as the scale effect dominates the technical and composition effects. It may be due to the reduced possibilities of further improving the diminishing returns or the sector-wise distribution based on technological advancements [72].

## 5 Conclusion

This study investigates the financial inclusion-CO2 emissions nexus for lower- and higher-income countries in an extended STIRPAT framework. The findings suggest that the N-shaped relationship exists between CO2 and inclusive financial systems in our total sample of 102 countries from 2004 to 2020. We further provide evidence suggesting variations across our subsamples of developed and developing countries in the N-shaped relationship. The findings for the subsample reveal substantial N-shaped nexus in developing countries, whereas a weak N-Shaped relationship in developed countries. Our results are robust across two normalized financial inclusion indices and alternate estimation techniques like FGLS and Differenced GMM.

Our findings on the N-shaped nexus suggest different phases of financial inclusion than the U-shaped EKC-based stages in the short, medium, and long run, as [19] found. Moreover, the differences are spread across developed and developing countries, implying that the stages may vary with the level of governance, regulations, and income. However, the findings on the N-shaped environmental effects of financial inclusion do not suggest reducing financial inclusion but require policymakers to facilitate well-directed and articulated financial inclusion policies. Specifically, policymakers need to consider setting up legal, governance, and regulatory policies that are more universal across countries while increasing the inclusive availability of climate finance to support developing countries in reducing carbon emissions. In addition, impetus and priority should be given to individuals and small & medium-sized enterprises in low-income countries regarding access to climate finance, enabling the adoption of technologies and processes to reduce GHG emissions.

Our study provides valuable insights into the financial inclusion-CO2 emission nexus and the importance of considering financial and environmental policies to address the issue. Overall, it is clear that reducing CO2 emissions will require sustained effort and cooperation from both developed and developing countries. This will require policy action, technological innovation, and a shift in how we think about energy and the environment. Based on our study’s findings, governments of both developed and developing countries should consider setting up universal legal, governance, and regulatory policies across countries while increasing the availability of climate finance to support developing countries in reducing carbon emissions. In addition, policies prioritizing individuals and small and medium-sized enterprises in low-income countries regarding access to climate finance could enable adopting technologies and processes to reduce greenhouse gas emissions.

Though we have thoroughly attempted to investigate the nexus between financial inclusion and CO2 emissions in a multivariate setting, we acknowledge the limitations of our study. First, our study only covers 2004 to 2020, which may not be enough to capture the relationship entirely. The study makes comparisons between lower-income and high-income countries, which may not fully reflect the complexities and variations within each country. Moreover, the study uses the STIRPAT framework and two normalized financial inclusion indices, which may not fully capture the complexities of our estimated model.

To overcome these limitations and enhance the findings of our study, future research may consider aggregating sample countries into regions such as Asia, Africa, America, and Europe in examining the N-shaped relationship between GHG emissions and inclusive financial systems. Future research may also investigate the impact of country-level governance on this nexus. Next, future research may consider developing the financial inclusion index based on a broad range of significant features, for instance, the accessibility and availability of the financial system for each country. Finally, other factors, such as technology, energy mix, economic structure, and policy, should also be employed in future studies to understand this nexus better.

## Author contribution statement

Shahzad Hussain and Raazia Gul; conceived and designed the experiments.

Syed Jawad Hussain Shahzad and Muhammad Akbar; performed the experiments.

Nader Naifar, Shahzad Hussain and Muhammad Akbar; analyzed and interpreted the data.

Shahzad Hussain, Raazia Gul, Syed Jawad Hussain Shahzad and Nader Naifar; wrote the paper.

## Data availability statement

Data will be made available on request.

## Declaration of competing interest

The authors declare that they have no known competing financial interests or personal relationships that could have appeared to influence the work reported in this paper

## Figures and Tables

**Table:** 

Total Sample (102 Countries)
Albania, Angola, Antigua and Barbuda, Argentina, Armenia, Austria, Azerbaijan, Bahamas, Bangladesh, Barbados,Belgium, Belize Benin,Bhutan, Bolivia, Bosnia and Herzegovina, Botswana, Brazil, Brunei Darussalam, Bulgaria, Burkina Faso, Burundi, Cabo Verde, Cambodia Cameroon, Canada, Central African Republic, Chad, Chile, China, Colombia, Comoros, Congo, Rep., Costa Rica, Croatia, Cyprus, Czech Republic Denmark, Djibouti, Dominica, Dominican Republic, Ecuador, Egypt, Arab Rep., El Salvador, Equatorial Guinea, Estonia, Eswatini, Ethiopia Fiji, Finland, France, Gambia, The Georgia, Germany, Ghana, Greece, Guatemala, Guinea, Guyana, Honduras, Hong Kong SAR, Hungary, Iceland, India, Indonesia,Iran, Islamic Rep., Iraq, Ireland, Israel, Italy, Jamaica, Japan, Jordan, Kazakhstan, Kenya Kiribati, Korea, Rep., Kuwait, Kyrgyz Republic, Latvia, Lebanon, Lesotho, Liberia, Libya, Liechtenstein, Lithuania, Luxembourg Macao SAR, China Madagascar, Malawi, Malaysia, Malta, Mauritania, Mauritius, Micronesia, Fed. Sts., Moldova, Mongolia, Morocco, Myanmar, Nauru, Namibia
Developed Countries (65 countries)
Antigua and Barbuda, Argentina, Armenia, Austria, Azerbaijan, Bahamas, Barbados, Belarus, Belgium, Belize, Bosnia and Herzegovina, Botswana, Brazil, United States, Brunei Darussalam, Bulgaria, Canada, Chile**,** China, Colombia, Costa Rica, Croatia, Cyprus, Czech Republic, Denmark, Dominica, Dominican Republic Ecuador, Equatorial Guinea, Estonia, Fiji, Finland, France, Georgia, Germany, Greece, Guatemala, Guyana, Hong Kong SAR China, Hungary, Iceland, Indonesia, Iran, Islamic Rep., Iraq, Ireland Israel, Italy, Jamaica, Japan, Jordan, Kazakhstan, Korea, Rep., Kuwait, Latvia, Lebanon, Libya Liechtenstein, Lithuania, Luxembourg, Macao SAR, China, Malaysia, Malta, Mauritius, Nauru, Namibia
Developing Countries (37 Countries)
Angola, Bangladesh, Benin, Bhutan, Bolivia, Burkina Faso, Burundi, Cabo Verde, Cambodia, Cameroon Central African Republic, Chad, Comoros, Congo, Rep., Djibouti, Egypt, Arab Rep., El Salvador, Eswatini, Ethiopia, Gambia, The Ghana, Guinea, Honduras, India, Kenya, Kiribati, Kyrgyz Republic Lesotho, Liberia, Madagascar, Malawi, Mauritania, Micronesia, Fed. Sts. , Moldova, Mongolia, Morocco, Myanmar

## Appendix A

Table A.1. Sample details.

## Footnotes

1 The Fifth Inter-Governmental Panel on Climate Change assessment report is available at: https://www.ipcc.ch/site/assets/uploads/2018/05/SYR_AR5_FINAL_full_wcover.pdf.

2 https://www.eea.europa.eu/highlights/eu-achieves-20-20-20.

3 https://www.epa.gov/climate-indicators/climate-change-indicators-us-greenhouse-gas-emissions.

4 https://chinapower.csis.org/china-greenhouse-gas-emissions/.

5 https://www.iea.org/reports/southeast-asia-energy-outlook-2022/key-findings.

6 https://www.worldbank.org/en/topic/financialinclusion/overview#:∼:text=Financial%20inclusion%20means%20that%20individuals,a%20responsible%20and%20sustainable%20way.
